# 10-Year Overview of the Hospital-Based Prevalence and Treatment of Congenital Cataracts: The CCPMOH Experience

**DOI:** 10.1371/journal.pone.0142298

**Published:** 2015-11-05

**Authors:** Duoru Lin, Jingjing Chen, Zhuoling Lin, Xiaoyan Li, Xiaohang Wu, Erping Long, Lixia Luo, Bo Zhang, Hui Chen, Wan Chen, Li Zhang, Haotian Lin, Weirong Chen, Yizhi Liu

**Affiliations:** State Key Laboratory of Ophthalmology, Zhongshan Ophthalmic Center, Sun Yat-sen University, Guangzhou, Guangdong, People´s Republic of China; Tsinghua University, CHINA

## Abstract

A review of 6 years of hospitalization charts from Zhongshan Ophthalmic Center (ZOC) revealed that congenital cataracts (CC) accounted for 2.39% of all cataract in-patient cases and that the age at surgery was decreasing before the establishment of the Childhood Cataract Program of the Chinese Ministry of Health (CCPMOH) in December 2010. We aimed to investigate data from the 4 years (January 2011 to December 2014) following the establishment of the CCPMOH, compared, and combined with data from the previous study period (January 2005 to December 2010) to generate a 10-year overview of the hospital-based prevalence and treatment of CC. In the 4-year period after CCPMOH establishment, the prevalence of CC was 2.01% in all hospitalizations, and was 2.78% in all cataract in-patients. Most of the eligible CC in-patients (71%) lived in south China. The ratio of boys to girls was 1.42:1. Nearly 2/3 of the patients underwent cataract extraction with primary intraocular lens (IOL) implantation at a mean age of 78.40±51.45 months, and cataract extraction surgeries without IOL implantation were performed in the remaining 1/3 of patients at a mean age of 10.03±15.92 months. After CCPMOH establishment, an increased incidence of CC was revealed, and the CC in-patients were younger than the patients in the previous period. The 10-year overview (2421 CC in-patients from 206630 hospitalizations) revealed upward trends in both the number and the prevalence of CC and a further reduction in age at surgery. In conclusion, the data from 4-year period after CCPMOH establishment and the 10-year overview showed upward trends in the hospital-based prevalence of CC cases and a further reduction in age at surgery, likely reflecting the effects of the CCPMOH establishment and providing useful information for further CC studies and a valuable foundation for the prevention and treatment of this cause of childhood blindness.

## Introduction

During the past decade, congenital cataracts (CC) have remained one of the chief factors leading to treatable childhood blindness.[1, 2] The global prevalence of this pathology has been shown to be 1 to 15 per 10000 children, and the prevalence is approximately 10-fold higher in developing countries than in developed countries.[3] Surgery is the first-line treatment, but the outcomes of operations are poor, with numerous comorbidities and severe intraoperative and postoperative complications, such as strabismus, nystagmus, posterior capsular opacity, and glaucoma.[4, 5] Furthermore, appropriate and timely treatment was restricted to developed areas due to the lack of equipment, techniques, and strategic guidance from the Ministry of Health, as well as to the lack of funding in poor regions.

Population-based data on epidemiology and surgical treatments are crucial for improving national prevention and treatment programs that address this challenging condition, especially in developing countries. However, to our knowledge, published reports of the prevalence and incidence of CC in China were unavailable prior to the establishment of the Childhood Cataract Program of the Chinese Ministry of Health (CCPMOH).[6] Investigation of the CC population at the Zhongshan Ophthalmic Center (ZOC), the base of the CCPMOH and one of the best, largest, and oldest eye hospitals using representative, current CC treatment methods in China,[7–9] is the best approach for improving this urgent situation because it is time-saving and inexpensive. In our previous study, a review of 6 years of hospitalization charts from the ZOC before the establishment of the CCPMOH in December 2010 revealed that CC accounted for 2.39% of all cataract in-patients and showed a decrease in age at surgery.[6] In the present study, we aimed to investigate the hospital-based CC prevalence and age at surgery in the 4 years following the establishment of the CCPMOH and to compare the two periods, which have similar lengths, in a 10-year overview of the prevalence and treatment of CC. This 10-year study of the epidemiology and surgical treatment of the large, representative sample of hospitalized CC patients in the ZOC could provide a useful reference for further studies of CC and, more importantly, for the development of national management strategies that advocate for the prevention of this cause of childhood blindness before any national epidemiological data are available.

## Materials and Methods

### Inclusion Criteria and Ethics Statement

Based on our previous study, we conducted another hospital-based, cross-sectional study of the hospital charts of CC patients over the 4-year period (from 1 January 2011 to 31 December 2014) following the establishment of the CCPMOH at the ZOC in Guangzhou, China. In the database of the Medical Records Department of the ZOC, which encodes cataract types and ocular abnormalities using the International Classification of Diseases, 9^th^ Revision, Clinical Modification (ICD-9-CM),[10] we identified CC hospitalizations with the following three codes: infantile cataract (366.0), congenital cataract and lens anomalies (743.3), and aphakia and other disorders of the lens (379.3). All CC in-patients who were treated surgically for the first time and who were ≤ 18 years old were included in a purpose-built database for analysis. Every case record was accepted and reviewed by two independent researchers to confirm the presence of CC in patients with or without other ocular abnormalities. Cataract extraction with or without primary intraocular lens (IOL) implantation constituted two different cataract surgery methods that were considered to be involved. As a key part of the CCPMOH, this study followed the tenets of the Declaration of Helsinki and was approved by the institutional review board of ZOC in Sun Yat-sen University (IRB-ZOC-SYSU), Guangzhou, China. All patient records and information were anonymized and de-identified before being analyzed, and our study was exempted from participant consent by the IRB-ZOC-SYSU.

### Information Extraction

All of the eligible data were carefully reviewed by two independent researchers. The incidence of CC, residence, gender, presenting age, laterality, hospitalization time, associated ocular abnormalities, and type of surgical procedure were extracted. Because of the retrospective nature of this study, information recorded in the medical records constituted the primary data, and some CC in-patients with incomplete data records were not included in the related analysis. Furthermore, it is worth noting that amblyopia was also not included in the analysis of ocular abnormalities because the children were too young for visual acuity and amblyopia assessments. As in the previous study, patients were divided into 5 groups according to age as follows: less than 6 months old (≤ 6 M), 6 months to 2 years old (6 M > and ≤ 2 Y), 2 to 6 years old (2 Y > and ≤ 6 Y), 6 to 12 years old (6 Y > and ≤ 12 Y), and 12 to 18 years old (12 Y > and ≤ 18 Y). Moreover, to determine the reasons for delayed hospitalization, patients ≥ 10 years old were chosen for further analysis. To better compare and combine the current results with those from the previous study period to obtain a 10-year overview, the data from the previous study were rearranged and reanalyzed according to the standards mentioned above.

### Statistical Analysis

All of the data were inputted into Microsoft Excel (Microsoft Corp., Redmond, Washington, USA) spreadsheets, sorted and analyzed by two researchers, and mutually checked. The data were also entered into the Statistical Package for the Social Sciences (SPSS ver. 19.0, Chicago, IL, USA) for statistical analysis. Absolute frequency (n) and relative frequency (%) were used for qualitative variables, such as the prevalence of CC and the ratio of boys to girls. The Pearson chi-square test was used for the comparison of the rates of CC, boys, native patients (from Guangdong province), unilateral and younger patients (≤ 6 years) between two periods before and after CCPMOH establishment. While the comparison of the age at surgery between periods was analyzed using T-test for independent samples. The level of significance was set at P < 0.05.

## Results

### The CC status 4 years after CCPMOH establishment

In the 4-year period after CCPMOH establishment, 90494 patients who were hospitalized for different eye diseases were recorded, including 65666 (72.56%) cataract patients and 1823 (2.01%) CC patients. Of these patients, 1193 were treated surgically for the first time, and the data for these patients were extracted and analyzed. The length of stay of most of the eligible CC patients was 1 (646, 54.15%) or 2 (471, 39.48%) days, with only 76 (6.37%) patients having hospital stays of ≥ 3 days. Of the eligible CC patients, 35.62% (425/1193) presented with a unilateral cataract, with the left eye and right eye involved in 19.53% (233/1193) and 16.09% (192/1193) of cases, respectively.

**Fig 1:**
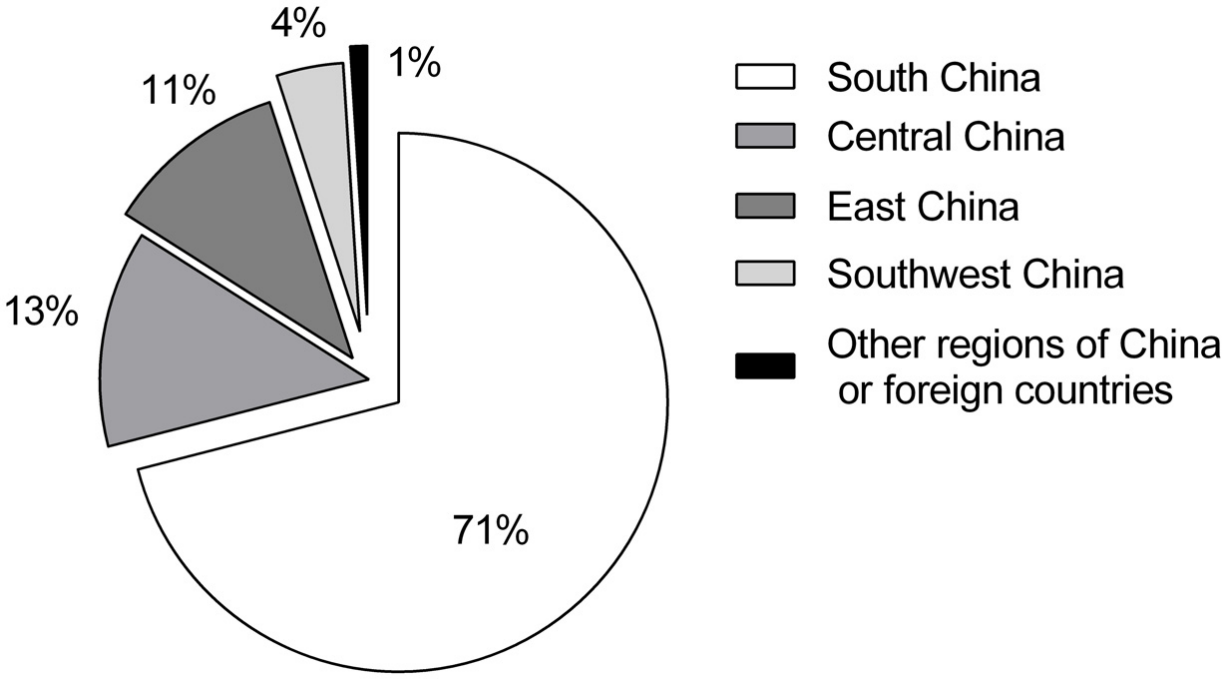
Residences of patients with congenital cataract hospitalizations. Most patients came from south China, while others came from other regions of China or from foreign countries.

**Fig 2:**
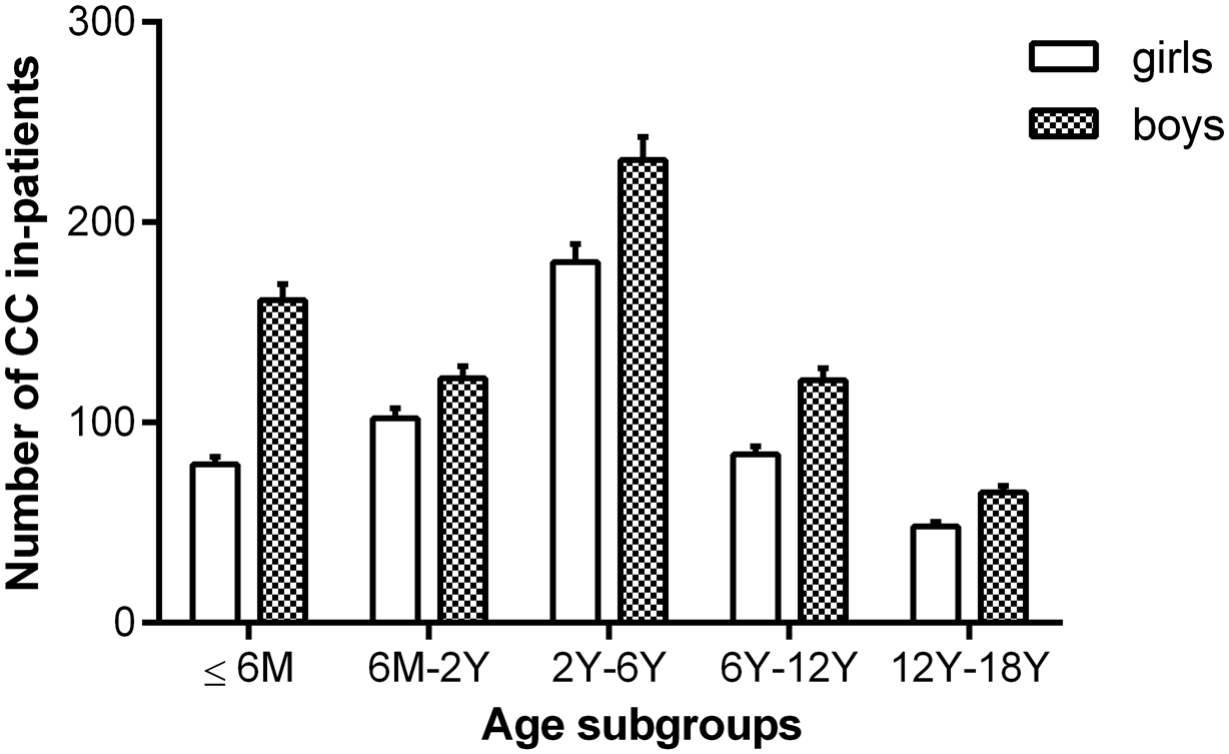
Number of CC in-patients in the age subgroups in the 4-year period after CCPMOH establishment. Patients 2–6 years old and 12–18 years old constituted the highest and lowest patient proportions, respectively. CC: congenital cataracts; M: months; Y: years.

According to the demographic analysis, most of the patients came from different regions of China or from foreign countries (Fig 1). Boys accounted for 58.68% of the patients, and the ratio of boys to girls was 1.42:1. Based on the age subgroup information, as shown in Fig 2, patients 2–6 years old and those 12–18 years old constituted the highest (34.45%) and lowest (9.47%) proportions of all groups of analyzed CC in-patients, respectively.

With regard to surgeries, nearly two-thirds of the CC in-patients (758/1193, 63.54%) underwent cataract extraction with primary IOL implantation at a mean age of 78.40±51.45 (95% CI 74.73 to 82.06) months, while cataract extraction surgeries without IOL implantation were performed on the remaining one-third (435/1193, 36.46%) of patients at a mean age of 10.03±15.92 (95% CI 8.53 to 11.53) months, 11.49% (50/435) of whom underwent IOL implantation in a secondary IOL implantation surgery 25.08±10.82 months later, before the end of this study.

The prognosis of CC patients would be significantly different if occurrences of other isolated ocular anomalies were accounted for. In the study period, except for 1009 patients (84.58%) without related records, 184 patients (15.42%) were confirmed to have one or more other ocular abnormities. Strabismus (exotropia and esotropia) was the most common ocular anomaly and was recorded in 70 CC in-patients (41 unilateral and 29 bilateral), with an incidence of 5.87%. The prevalence of nystagmus was 3.60% (43/1193), which accounted for the second most frequent ocular abnormality. The third most common ocular anomaly in our study was refractive error (41/1193, 3.44%), including myopia, high myopia, and anisometropia. Anomalies of the vitreous (persistent hyperplastic primary vitreous (PHPV) and vitreous opacities) had a prevalence of 2.01% (24/1193) and constituted the fourth most common ocular abnormality. Anomalies of the lens (0.67%, 8/1193), congenital microcornea (0.50%, 6/1193), and congenital microphthalmia (0.25%, 3/1193) were the following three most common ocular anomalies according to the medical records in our study. In addition, apart from these ocular anomalies, two cases of Down's syndrome were also noted and were probably associated with CC.

**Fig 3:**
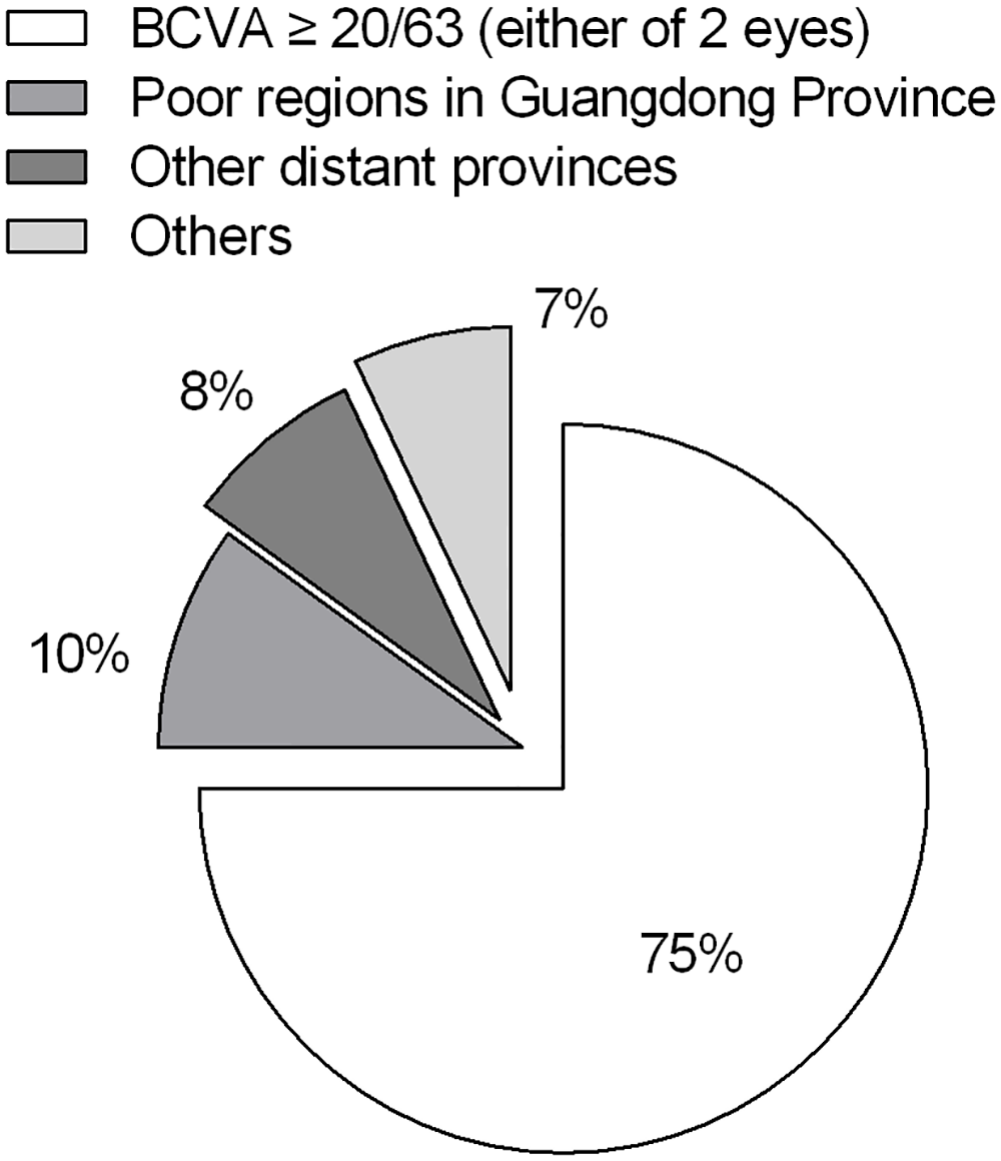
Reasons for delayed surgical treatment of patients ≥ 10 years old. The leading cause was relatively good preoperative visual acuity. Poverty (poor regions in Guangdong Province) and long distance (other distant provinces) were the following main factors. BCVA: best corrected visual acuity.

To better analyze the reasons for delayed hospitalization, 151 CC patients ≥ 10 years with complete data underwent further analysis. The results of this analysis are shown in Fig 3. The best corrected visual acuity (BCVA), evaluated in 74.83% (113/151) of these patients, was 20/63 or better. Of the rest, 86.84% (33/38) of the patients with a BCVA worse than 20/63 were either from poor regions in Guangdong Province (31.58%, 12/38, bottom 1/3 GDP in 2014) or other distant provinces (28.95%, 11/38).

### Comparison of the two periods before and after CCPMOH establishment

For the purpose of accurate comparison, the data from the previous study period (from 1 January 2005 to 31 December 2010) were rearranged and reanalyzed according to the same standards used for this analysis, which was based on data obtained after CCPMOH establishment. The change in CC prevalence and the treatments administered before and after CCPMOH establishment are listed in Table 1. These data demonstrate a larger number of CC in-patients (per year) after CCPMOH establishment, most of whom were boys and came from Guangdong Province. However, the ratios of boys and of patients from Guangdong Province markedly decreased compared with those in the previous period. Furthermore, CC in-patients after CCPMOH establishment were found to be younger than those before its establishment.

**Table 1:** Comparison of the two periods before and after CCPMOH establishment.

	6Y period before	4Y period after	Pearson χ^2^	P
Total in-patients (/Y)	19356	22624	-	-
Cataract in-patients (/Y)	9020	16417	-	-
CC in-patients (/Y)	327	456	-	-
CC prevalence (%)	1.69	2.01	30.23	< 0.01*
Boys: girls	1.72:1	1.42:1	5.38	0.02*
Guangdong Province (%)	73.62	65.13	20.53	< 0.01*
Unilateral CC in-patients (%)	31.92	35.62	3.71	0.05
Age (mean ± SD, months)	61.79±58.45	53.47±53.45	-	< 0.01^#^
CC in-patients ≤ 6Y (%)	67.10	73.34	11.27	< 0.01*
Cataract extraction (%)	32.90	36.46	3.39	0.07

Y: year or years; CC: congenital cataracts; CC prevalence: the ratio of CC in-patients to total hospitalizations.

*: Pearson chi-square test, and P < 0.05, statistically significant;

^#^: independent sample T-test, and P < 0.05, statistically significant.

### 10-year overview of the hospital-based prevalence and treatment of CC

**Fig 4:**
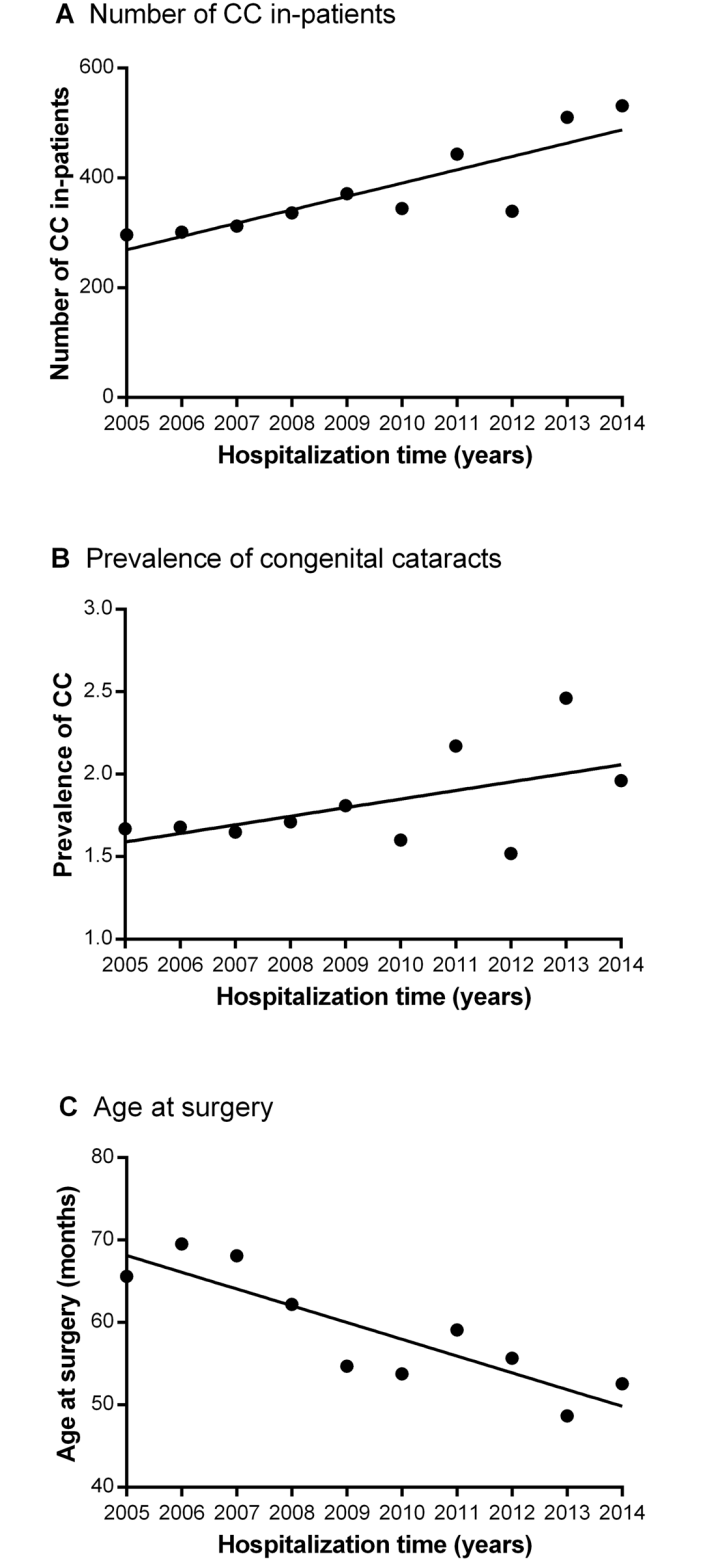
10-year overview of the hospital-based prevalence and treatment of CC. (A & B) The number of CC in-patients and the incidence of CC increased during the 10-year period. (C) A downward trend in age at surgery was observed during this decade. CC: congenital cataracts; Prevalence of CC: the ratio of CC in-patients to total hospitalizations.

The data used for the 10-year overview of the prevalence and treatment of CC, shown in Fig 4, were available to compare and combine with the rearranged data from the previous study period (a total of 2421 CC in-patients from 206630 hospitalizations were included in the analysis). The results revealed upward trends in both the number (panel A) and prevalence (panel B) of CC in this 10-year study period, as well as a reduction in the age at surgery (panel C).

## Discussion

CC is among the primary causes of treatable childhood blindness, which severely affects the health of children worldwide. The prognosis for these patients is extremely poor because of the particular nature of CC pathology. Even worse, the treatment for this rare disease is available to only a small proportion of people and deserves greater attention from national health care departments. Population-based data on epidemiology and surgical treatments are critical for improving management strategies for this challenging condition. To better assess and strengthen the management of CC, the CCPMOH was founded in 2010 in ZOC. The hospital-based epidemiological data and age at surgery of CC in-patients were recently published in a review of 6 years of hospitalization charts from ZOC before CCPMOH establishment.[6] Based on our previous work, we investigated the following 4 years of data, after CCPMOH establishment, and compared the two similar-length periods within a 10-year overview of the hospital-based prevalence and treatment of CC. In the 4-year period after the establishment of the CCPMOH, CC accounted for 2.78% of cataract in-patients and 2.01% of total hospitalizations. The age at surgery was 78.40 months for patients undergoing cataract extraction with primary IOL implantation and 10.03 months for those undergoing cataract extraction without IOL implantation. A larger number of CC in-patients (per year) were revealed to have been treated and diagnosed and an increased incidence of CC was found after CCPMOH establishment, while the proportions of boys and patients from Guangdong Province were decreased compared with the previous 6-year period, and the CC in-patients were younger after CCPMOH establishment than before. This 10-year overview reveals an upward trend in both the number of cases and the prevalence of CC, as well as a reduction in age at surgery.

The prevalence of CC is approximately 5.0 per 10000 births in China, as has been shown in some available small-population studies.[11] Recently, it was reported that the CC incidence was 0.1% among 9512 pupils in a study of the prevalence and causes of visual impairment in schools for the children of migrant workers in Shanghai, China.[12] The current study is different from these earlier studies in that this was a 10-year, large scale and hospital-based serial study, and it is the first report on the overall prevalence of CC in south China. We found that CC accounted for 2.39% of all cataract in-patients in a review of 6 years of hospitalization charts from ZOC prior to the CCPMOH establishment, and this percentage increased to 2.78% in the following 4-year period. This discovery probably reflects the maximum prevalence of CC in the broader population of China. Furthermore, increasingly more CC patients who presented to ZOC were from all parts of the country other than the native province (Guangdong Province) after CCPMOH establishment. The increased number of new and non-local CC patients in the following 4-year period could perhaps have benefited from the enlargement of the scope of screening and the strengthening of the intensity of financial aid after CCPMOH establishment.[7, 8] A relatively small population (314 patients) study in Iran revealed that boys were almost 10% more frequently treated than girls.[13] We demonstrated a similar treatment ratio of boys to girls (1.72:1) in a large number of CC in-patients in our previous study, which was greater than the Chinese population gender ratio (male: female = 51.27:48.73%). The high proportion of boys with CC may be attributed to the traditional preference for sons in China or to gender-related genetic mechanisms that have previously been described.[9] Interestingly, the ratio dropped to 1.42:1 in the later 4-year period, which is probably related to changes in gender perception and sexual equality in modern society.

The age at surgery is a crucial success factor for the prognosis of CC patients. To prevent CC patients from developing irreversible deprivation amblyopia, early diagnosis and timely surgery during the critical period of visual development are extremely important.[14] Most CC patients can be identified and treated within 100 days of birth in many developed countries because of national screening procedures,[14–16] whereas the mean age at operation for CC patients in some developing countries is 3.2 years old (38.4 months).[13] In China, we revealed in our previous study, for the first time, the age at surgery (27.62±23.36 months) of a large sample of CC patients aged no more than 6 years old. For a better comparison, we then analyzed the age at surgery of CC in-patients ≤ 18 years old in the two similar-length periods before and after CCPMOH establishment within this 10-year overview. The results showed that the average age at surgery decreased from 61.79 months (in the 6-year period before CCPMOH establishment) to 53.47 months (in the later 4-year period). The mean age of patients who underwent cataract extraction was 10.03 months in the later period, which was much younger than that of the patients who underwent cataract extraction and IOL implantation (78.4 months). In addition, the proportion of patients who were no more than 6 years old increased significantly after CCPMOH establishment, indicating a downward trend of age at surgery resulting from the effort made by CCPMOH. This tendency may also be attributed to the strengthened recognition of the significance of early treatment for CC by parents and pediatric ophthalmologists and to the continuing improvement of ophthalmic surgical techniques and anesthetic techniques in China.[7, 8] However, delayed presentation to the hospital and late surgical treatment are still common in China compared with developed countries.[11] To determine the reasons for delayed hospitalization, patients ≥ 10 years were identified for further analysis. We found that the leading cause was relatively good preoperative visual acuity, while poverty (poor regions in Guangdong Province) and long distance (other distant provinces) were the following main factors. These findings provide valuable information for the development of national medical science policies that advocate for the timely treatment of this cause of childhood blindness and for the prevention of visual impairments. Apart from the timing of surgery, it has also been reported that the presence of other associated ocular abnormalities, such as nystagmus, strabismus, PHPV, small eyes, and small corneal disorders, also has a significant effect on prognosis.[17, 18] In the 4-year period after CCPMOH establishment, the top three most common ocular abnormalities found in CC in-patients, namely, strabismus (exotropia and esotropia), nystagmus, and refractive error, were similar to those reported in our previous study and in other studies. For CC patients who present complications related to associated ocular abnormalities, a careful assessment and detailed explanation for possibility of poor postoperative visual function is needed.

One limitation that should be noted is that because this was a retrospective study, some deviations in data entry from information recorded in medical records are likely. Another limitation is probable bias in patient recruitment, as the records used do not provide a complete representation of the population but only of surgical patients at the best eye center in south China. The third limitation is the different analysis criteria used between the two related studies; however, this issue was largely resolved by the rearrangement and reanalysis of the data from the previous report according to the standards used for the current study. Notwithstanding these limitations, this relatively large population-based study providing a 10-year overview does yield valuable information regarding the hospital-based prevalence of and treatment tendencies for CC over the most recent decade in China, prior to the publication of official data in national surveys, which is time-consuming and costly, and reveals a significant improvement in CC management strategies after CCPMOH establishment.

In conclusion, the present study demonstrated a larger number of CC in-patients and an increased incidence of CC after CCPMOH establishment. The 10-year overview also revealed upward trends in the hospital-based prevalence of CC, as well as the further reduction of the age at surgery, which probably reflects the effects of the CCPMOH, and provides useful information for further CC studies and a valuable foundation for the prevention and treatment of this cause of childhood blindness.
